# An Overview of Abiotic Stress in Cereal Crops: Negative Impacts, Regulation, Biotechnology and Integrated Omics

**DOI:** 10.3390/plants10071472

**Published:** 2021-07-19

**Authors:** Rajendran Jeyasri, Pandiyan Muthuramalingam, Lakkakula Satish, Shunmugiah Karutha Pandian, Jen-Tsung Chen, Sunny Ahmar, Xiukang Wang, Freddy Mora-Poblete, Manikandan Ramesh

**Affiliations:** 1 Department of Biotechnology, Science Campus, Alagappa University, Karaikudi 630003, India; jeyasri8220@gmail.com (R.J.); pandianmuthuramalingam@gmail.com (P.M.); lsatish@post.bgu.ac.il (L.S.); pandiansk@gmail.com (S.K.P.); 2 Department of Biotechnology, Sri Shakthi Institute of Engineering and Technology, Coimbatore 641062, India; 3 Department of Biotechnology Engineering, Ben-Gurion University of the Negev, Beer Sheva 84105, Israel; 4 Department of Life Sciences, National University of Kaohsiung, Kaohsiung 81148, Taiwan; jentsung@nuk.edu.tw; 5 Institute of Biological Sciences, University of Talca, 2 Norte 685, Talca 3460000, Chile; sunnyahmar13@gmail.com; 6 College of Life Sciences, Yan’an University, Yan’an 716000, China; wangxiukang@yau.edu.cn

**Keywords:** abiotic stress, GWAS, *Oryza sativa* L., plant omics, *Triticum aestivum* L., *Sorghum bicolor* L., transcription factors, *Zea mays* L.

## Abstract

Abiotic stresses (AbS), such as drought, salinity, and thermal stresses, could highly affect the growth and development of plants. For decades, researchers have attempted to unravel the mechanisms of AbS for enhancing the corresponding tolerance of plants, especially for crop production in agriculture. In the present communication, we summarized the significant factors (atmosphere, soil and water) of AbS, their regulations, and integrated omics in the most important cereal crops in the world, especially rice, wheat, sorghum, and maize. It has been suggested that using systems biology and advanced sequencing approaches in genomics could help solve the AbS response in cereals. An emphasis was given to holistic approaches such as, bioinformatics and functional omics, gene mining and agronomic traits, genome-wide association studies (GWAS), and transcription factors (TFs) family with respect to AbS. In addition, the development of omics studies has improved to address the identification of AbS responsive genes and it enables the interaction between signaling pathways, molecular insights, novel traits and their significance in cereal crops. This review compares AbS mechanisms to omics and bioinformatics resources to provide a comprehensive view of the mechanisms. Moreover, further studies are needed to obtain the information from the integrated omics databases to understand the AbS mechanisms for the development of large spectrum AbS-tolerant crop production.

## 1. Introduction

Cereals are grasses (a monocot family Poaceae, also known as Gramineae) cultivated for the edible components of the grain. The most important staple cereal crops are wheat, rice, maize, sorghum, barley, oats, and millet. These cereals are cultivated for the edible components of their caryopsis, composed of the endosperm, germ, and bran. These plants have evolved to live in environments where they are often exposed to various stressors such as high temperature (HT), drought, salinity and mineral toxicity, and the water deficiency [1,2]. Cereals are widely utilized crops in world agriculture, with an overall production of 2500 million tonnes being harvested globally in 2011. On a worldwide basis, rice, wheat and maize are the three most important cereal crops, which together comprise at least 75% of the world’s grain production. Also, in 2011 723, 704, and 883 million tonnes of rice, wheat, and maize were harvested, respectively. Cereals contain major nutritional and energy sources such as proteins, carbohydrates, minerals, amino acids, fiber, and micronutrients such as vitamins, magnesium, and zinc for the global population [3,4]. Asia, America, and Europe produce 80% of the world’s cereal grains. Rice, sorghum, millet and wheat are widely produced in Asia; likewise, corn and sorghum in America and barley, rye, and oats in Europe. Cereals are a pivotal nutrient source in both developed and developing countries, however, the utilization pattern of these cereal grains differs. In developed countries, more than 70% of total cereal production is fed to the animals, whereas in underdeveloped countries, 68 to 98% of the cereal production is used for human consumption [5].

Abiotic stresses (AbS) (predominantly drought, cold, salinity, and heat), adversely affect diverse plant developmental stages. They are highly complex and affect the various plant dynamisms at the transcriptome, cellular, and physiological processes such as flowering, grain filling, and maturation [6,7]. The ability of cereal crops to tolerate dominant AbS comprises water deficit (drought), flood (anoxia), salinity, high/low temperature, and other osmotic stresses is an essential aspect of yield resilience, and its improvement has long been a target for plant breeders and researchers [8]. The rapidly changing global climate is affecting crop productivity and food availability due to the ever-increasing population, resulting in a demand for stress-tolerant crop varieties that have never been greater [9,10]. Understanding the molecular cross-talk of plant responses to various stresses is crucial in providing opportunities for the development of broad-spectrum stress-tolerant crops. As a result, it is crucial to understand the AbS tolerance dynamic while also devising a new and improved approach for dealing with their detrimental influence on the agricultural sector. The recent game-changing advances in bioinformatics and integrating omics technologies could serve as the most immediate and prospective strategies for improving AbS tolerance in cereal crops. Omics approaches lead to understanding the stress tolerance mechanisms at the molecular level including genomics, functional genomics, genetic engineering, gene expression, protein or metabolite profile(s), and their overall phenotypic effects. In crop breeding techniques, the identification and characterization of the genes and the specific genetic regions associated with both the quantitative and qualitative agronomic traits have been a major challenge. In recent breeding programs, a high-throughput marker-assisted system is extensively being used to enhance selection accuracy and efficiency.

## 2. Cereal Crops

### 2.1. Comparative Nutritive Values of Cereal Crops

Cereal grains have low protein content when compared to food legumes and oilseeds, with rice being the lowest. Among the essential amino acids for humans, lysine is the most limiting in all cereal grains. Most cereal proteins are rich in cysteine, methionine and sulfur-containing amino acids. The biological value (BV) ranges from 55 to 77.7%, protein digestibility (TD) 77 to 99.7%, and net protein utilization (NPU) 50 to 73.8% in different cereal diets fed to growing rats. Barley contains relatively more amounts of lysine compared with other cereal crops. The utilization of legumes is known to be affected by the presence of several antinutrients, such as metal chelates, antivitamins, goitrogens, cyanogens, inhibitors of proteases and amylases, toxic phenolic glycosides, and amino acid derivatives [11]. Hence, proper processing of cereal-legume mixture is required to minimize these antinutrients before consumption. Compared with animal foods cereal grains products are inferior in both nutritional and sensory qualities. Physical, chemical, biological, and/or physiological modifications can improve both the nutritional and evident qualities of the grains [12]. Moreover, natural processes like fermentation and controlled germination with natural microflora are highly beneficial in improving the quality of cereal-based food. Important cereal crops and their comparative nutritive values are given in Table 1.

### 2.2. Rice (Oryza sativa *L.*)

Rice is the second most widely consumed cereal, serving as a staple food for more than half of the world population and 90% of Asians. Rice, known as the grain of life, contains 80% carbohydrates, 7–8% protein, 3% fat, and 3% fiber [13]. In most countries, rice being the most dominant cereal crop can improve the health condition of millions of people who consume it. It plays an important role in health benefits and Lifestyle-related disease prevention such as high blood pressure, cancer prevention, Alzheimer’s diseases, skincare, diabetes, heart disease, and dysentery [14,15,16]. However, rice plants are severely affected by various AbS, major stressors such as drought, cold, salinity, and high temperature [17].

Excess salinity in the soil is one of the major abiotic stress factors that affect the growth and productivity of a wide variety of crops including rice. Generally, rice can tolerate a modest amount of saltwater without affecting its growth and yield. However, it highly depends on the types and species of rice used as well as their growth stage [18]. According to Lee et al. [19], indica has a higher tolerance level than japonica at the seedlings stage. Rice is grouped as a salinity-sensitive cereal at an early stage of growth, which limits its production efficiency at the mature stage [20,21,22].

### 2.3. Maize (Zea mays *L.*)

Maize or corn is an important cereal crop grown in diverse agro-ecological zones and farming systems and socio-economic backgrounds in sub-Saharan Africa (SSA) [23]. After rice and wheat, maize is the world’s third most important crop, and it is known as the “Queen of Cereals” because it has the highest production potential of all the cereals [24]. It is a predominant source of nutrition as well as phytochemical compounds such as carotenoids, phenolic compounds, and phytosterols [25]. Phytochemicals are naturally occurring bioactive compounds in plants that provide human health benefits while also preventing the risk of major chronic diseases [25,26]. Maize is believed to have potential anti-HIV activity due to the presence of *Galanthus nivalis* agglutinin (GNA) lectin or GNA-maize [25]. The studies revealed that phytochemicals in grains due to their potent antioxidant activities demonstrate significantly reducing the risk of many diseases such as cardiovascular disease, type 2 diabetes, Diet-related disorders, and cancers [27].

The growth and productivity of maize are severely affected by several abiotic stresses such as salinity drought, waterlogging, cold, and nitrogen stress. Among them, salinity stress causes several biochemical and physiological changes in maize, such as disruption of cellular homeostasis, ionic imbalance, nitrogen fixation, respiration, inhibition of several metabolic enzymes such as photosynthetic enzymes etc (enzyme toxicity) [28]. Drought is one of the most detrimental AbS which are seriously affecting the productivity of cereal crops. However, while maize is known as the “Queen of Cereals” across the world, it is susceptible to drought. Drought can affect kernel weight following silking stage up to maturity. Severe drought can reduce the yields of maize during this period by 20 to 30%. During the grain filling stage because of reduced photosynthesis accelerated leaf senescence takes place [29]. More recently, new biotechnological tools have emerged to further accelerate the grain selection and improvement for enhancing the tolerance to drought conditions.

### 2.4. Wheat (Triticum aestivum *L.*)

Wheat is a major staple crop for several countries, grown on around 10 million ha in Africa and it comes from a type of grass (*T. aestivum* L.) that is grown in countless varieties worldwide. As a result of a growing population, changing food preferences, and socioeconomic change associated with urbanization and industrialization, wheat consumption steadily increased during the past two decades in all African countries [30,31]. Wheat consumption provides up to 50% of daily calories and proteins. Wheat is often considered to be a source of energy (carbohydrate) and also contains a significant amount of other important nutrients including proteins, fiber, and minor components such as lipids, vitamins, minerals, and phytochemicals that may significantly contribute to the individual diet [32,33]. It reduces the risk of cardiovascular disease, type 2 diabetes, and forms of cancer (notably colorectal cancer) [34,35]. Dietary fiber components also have high heritability and are amenable to manipulation by breeding. Therefore, plant breeders should be able to select plants with enhanced health benefits in addition to increased crop productivity [36,37].

However, wheat plants are severely affected by different AbS, such as salinity, drought, cold, and heat. Heat stress affects wheat growth and yield, particularly at grain developmental stages. The effect of a 3-day heat shock on biomass production was less than the pre- and post-treatment growing temperature [38]. Salinity stress also affects the growth and productivity of wheat. The increasing salinity of irrigation water had a significant adverse effect on the yield of wheat. Domestic wheat lines could be grown at salinity concentrations ranging from 4 and 8 g/L with minimal reduction in biological and grain yield. Moreover, many physiological and biochemical approaches have been developed in plants to survive at high salt concentration. The most effective approach to solve the salt problem is to improve wheat adaptation under salinity stress conditions and enhance its grain yield. Various biotechnological approaches are required to understand the genetic and physiological mechanisms of natural differences in salinity tolerance of wheat and to obtain methods to explore the inherent genetic differences, to get new candidate genes for improving salt tolerance in wheat [39,40].

### 2.5. Sorghum (Sorghum bicolor *L.*)

Sorghum is the fifth most important food and feed crop in the world. It is the main cereal food for semi-arid tropical regions of Africa, Asia, and Latin America. Sorghum species (*S. vulgare* and *S. bicolor*) are members of the grass family. Sorghum is resistant to drought and water-logging and is grown in different soil conditions [41]. It is mainly composed of starch, protein and unsaturated fatty acids and is an essential source of some vitamins and minerals [42]. Sorghum is the most abundant and omnivorous cereal secondary metabolites of plants, including up to 6% of 3-deoxyanthocyanidine, phenolic acid, flavonoids, and tannins [43,44]. Phenolic and soluble compounds play an important role in balancing or stabilizing the intestinal microbiota and the parameters associated with obesity, oxidative stress, inflammation, diabetes, dyslipidemia, hypertension, and cancer [45].

Among the various AbS, drought and temperature stress are of foremost important that limits sorghum production. Severe drought causes considerable yield loss in sorghum, and it has a greater impact on grain filling and flowering compared to vegetative stage. Drought adversely affects various physiological functions, inflorescence development and leaf growth of the sorghum [46,47]. Interestingly, genome and transcriptome sequencing, annotation projects and recent literatures paved the way to identify the candidate genes through omics databases, which are predicted to be involved in individual and combined abiotic stress (CAbS) responses. This claim supports to understanding the stress tolerance mechanism and environmental adaptation, as well as promoting the green revolution of all other food crops [47,48].

## 3. Abiotic Stress (AbS) Dynamism on Cereal Crops

The atmosphere, soil, water and their associated factors are the major abiotic stressors affecting modern agricultural systems [49].

### 3.1. Atmospheric Factors

#### 3.1.1. Rainfall

In semiarid regions, rainfall is one of the primary AbS factors affecting soil erosion and crop production in rain-fed agriculture. It controls soil salinity and acidic properties. Sulfur dioxide (SO_2_) and nitrogen oxides (NOx) from fossil fuel burning merge with water and oxygen in the atmosphere resulting in acid rain. It also reduces the soil pH and removes nutrients and minerals from the soil that can be harmful to plants [50].

#### 3.1.2. Temperature

The plant underwent a high temperature that caused certain mechanical damages including expansion-induced-lysis, phase transitions and fracture lesions in membranes, and physical damage [51]. Depending on the temperature plant species have been classified into three groups: chilling-sensitive, freezing-sensitive, and freezing-resistant plants [52]. Freezing may alter the growth and can cause frost-hardening/cold hardening and also induces the production of reactive oxygen species (ROS), which damages membrane components, and results in protein denaturation [53,54]. Chilling stress including reduced leaf expansion and wilting, affects the reproductive development of plants, chlorosis, and may lead to necrosis.

#### 3.1.3. Gases

An increase in the level of greenhouse gases in the atmosphere such as carbon dioxide (CO_2_), methane (CH_4_), nitrous oxide (N_2_O), ozone (O_3_), water vapor (H_2_O), and some artificial chemicals such as chlorofluorocarbons (CFCs) increases the atmospheric temperature. As CO_2_ levels increase in the atmosphere, nitrogen concentration is decreased in plants. Thus, decreasing the protein levels and affects the growth ability of plants [55].

#### 3.1.4. Radiation

Ultraviolet (UV) and ionizing radiations affect plant growth and development in many ways. Radiations may disrupt stomatal resistance, damage plant cells, increases cell mutations, prevents seed growth, and reduce plant fertility [56,57]. Photon irradiation (such as H_2_O, CO_2_, CH_4_, N_2_O, and O_3_) generates cellular damages in root and leaf tissue.

#### 3.1.5. Wind

Plant tissues can be damaged during hot, humid, hazy weather with little wind. Wind decreases the phytohormonal content of roots and shoots in cereal crops. The direction and velocity of the wind will affect plant growth and development [49].

### 3.2. Soil Factors

#### 3.2.1. Soil Properties

Salt stress (salinity) causes multifarious effects in plants such as ionic effect, osmotic effect, nutrient and hormonal imbalance, and production of ROS [58]. Plant growth and productivity are severely affected by the accumulation of sodium (Na^+^) and chloride (Cl^–^) ions that leads to creating ionic, osmotic, and oxidative stresses [59,60,61,62,63]. Sodium-ion (Na^+^) has been detected to intervene in multiple metabolic processes such as protein translation, transcription, and enzyme activity that ultimately led to osmotic stress. Functional genomic approaches provide new opportunities to unravel the functional roles of AbS responsive genes, enabling the identification of genes and generation of stress-tolerant plants especially on important cereal crops [64]. Plants can generally tolerate salinity stress mainly by three mechanisms, which include ion exclusion, osmotic tolerance and tissue tolerance. Osmotic tolerance is regulated by long-distance signaling waves that reduce cell expansion in root tips, leaves, and stomatal conductance [65,66]. Ion exclusion mainly involves the transport of Na^+^ and Cl^−^ into roots which prevent the reduction of Na^+^ accumulation in shoots. Tissue tolerance involves the exposure of tissues to the accumulated Na^+^ and Cl^−^ at cellular and intracellular levels, synthesis of compatible solutes, and production of the enzyme catalyzing detoxification of ROS [67].

#### 3.2.2. Pollution

Soil contamination was widespread as a result of the rapid application of some harmful pesticide compounds which can infiltrate the natural environment in two ways depending on their solubility. The pesticide-contaminated soil may affect the availability of nutrients in plants. Rapid industrialization near agricultural lands may affect crop growth and production. Heavy metals such as Fe, Mn, Zn, Cu, Mg, Mo, B, and Ni in the soil of a particular area significantly affect the morphological, metabolic, and physiological anomalies in plants. It ranges from chlorosis of shoot to lipid peroxidation and protein degradation [68]. Among them, ‘B’ is an essential element for plant growth and simultaneously affects the growth and yield at higher levels.

#### 3.2.3. Degradation

Nutrient deficiency has been considered as the main cause of poor crop productivity. Of the global land area of 13.5 billion ha, among that only 3.03 billion ha (22%) is cultivable and about 2 billion ha is degraded. Disposal of oil shales, heavy metal contamination of soil, and spillage of crude oils adversely affect the severe root damage that governs nutrient irregularity [69].

### 3.3. Water Factor

#### 3.3.1. Suboptimal

The most important constraints for agriculture are the water limitation, declining rainfall, and increasing temperature which significantly affects the growth of crop plant areas. A wide range of strategies such as genetical, physiological, biochemical, and molecular levels are well defined in plants. However, recent advancements are also available to obtain important drought-tolerant crops using conventional, marker-assisted breeding, and genetic engineering [70,71].

#### 3.3.2. Supraoptimal Salinity

High salinity can be toxic for many crops, but halophytes have adapted to the worse salt conditions, which are found in several coastal areas, such as salt marshes, and inland arid areas. These zones, having a high rate of evaporation tends to concentrate salts in the mineral content of the soil. Those halophytes are morpho-physiologically and reproductively adapted to saline, waterlogged and anaerobic conditions. Nutrient deficiency or the presence of toxic substances such as heavy metals in the soil can also result in the AbS [49].

#### 3.3.3. Waterlogging

Flood water usually causes a water-logged situation in the field. In waterlogged soils, within 24 h the oxygen concentration drops down to zero because water replaces most of the air in the soil pore space. Roots need oxygen for their respiration and cell viability. Water-logging limits oxygen supply to the roots, if any remaining oxygen is used up by the roots from flooded or waterlogged soils, then the normal function of the roots will be arrested. Therefore, the leaves and stems are unable to obtain enough minerals and nutrients hence the roots start to die off in water-logging conditions [72].

## 4. Bioinformatics and Functional Omics Approach to Explore the AbS Tolerance Mechanism

The process of gene regulatory dynamism in various cellular, physiological and biochemical mechanisms in plants was altered by environmental factors. Therefore, to analyze the processes involved in this regulatory mechanism, several functional omics projects, have been initiated across the world in recent years [49,73,74]. Moreover, multiple omics and bioinformatics approaches have been used for developing crop plants that are tolerant to AbS through molecular breeding and genetic engineering and also through advancements and increasing knowledge in genetics, genomics, and molecular physiology [75,76]. Hence, the development of new functional omics and computational biology software and tools paved the way to identify the AbS responsible candidate genes from gene pools. In addition, the use of high-throughput techniques has been employed such as expression reads by RNA-Seq, random and targeted mutagenesis, gene shifting, complementation, and synthetic promoter trapping approaches make many avenues for functional analyses of AbS responsive genes and tolerance mechanisms [77]. Transcription factors (TFs) are crucially important in knowing the appropriate molecular processes and pathways that are involved in plant growth and survival under AbS conditions [78,79,80]. AbS are the quantitative and multiple genes associated in nature, these stress molecular cross-talks and their pathway interconnections are found under AbS conditions. Again, understanding the post-translational modifications (PTM) degradation of proteins and non-coding miRNA interactions allow the modulation of the target proteins. Similarly, some of the siRNAs play an important role as stress-inducers and affect protein synthesis including alternative splicing. The genome-wide association studies (GWAS) have become popular to provide novel strategies to identify and characterize the unique stress-responsive genes, which are introduced into crop plants and used in building up the tolerance against various AbS conditions [80]. In this record, the identification, and characterization of specific stress-responsive genes along with their promoters are analyzed for specificity. Integrated omics and bioinformatics approaches have been used to study the AbS responsible genes, their corresponding growth regulations and their encoding global metabolic network. With the advent of newly developed functional omics can be broadly categorized into two potential approaches used in manipulating gene-pool for enhancing the AbS tolerance, which contains: (i) identification of stress-responsive genes followed by genetic engineering to improve the stress tolerant cereals development and (ii) mining of marker associated with agronomically important genes and their use in marker-assisted breeding programs.

Importance of Omics in Enhancing Nutritive Values

In the era of post-genomics, revealing the interconnected functional attributes between genes transcripts, metabolites, proteins and nutritional biology remains a major challenge. New advances in omics such as genomics, transcriptomics, proteomics and metabolomics will have an essential advantage in the bio-fortification processes in plants.

## 5. Gene Mining

Functionally integrated omics and computational biology tools for AbS tolerance include reconnaissance of novel AbS responsive genes and the expression levels, that may induce the AbS response. These omics approaches are used to understand the functional role of AbS -responsive genes and the generation of stress-tolerant transgenic lines. In addition, they also pave the way for guiding genetic and metabolic engineering studies. In cereals, large numbers of expressed sequence tags (ESTs) have been generated from different cDNA libraries, collected from AbS treated tissues at different developmental stages, and are considered as a significant functional genomic approach to impute the AbS responsive genes. The expressed sequence tag database (dbEST) provides information about the number and type of different AbS tolerant species. Genomics-based approaches were started in all the crop plants such as barley, rice, maize, wheat, and sorghum (stress-genomics.org). A total of 13,022 AbS related ESTs were reported from *Hordeum vulgare*, 13,058 genes from *O. sativa*, 17,189 from *S. bicolor*, 2641 from *Secale cereale*, 20,846 from *T. aestivum* and 5695 regulators from *Z. mays* using gene index of TIGR database (http://www.tigr.org/tdb/tgi/ accessed on 16 April 2021) [81]. However, publicly available ESTs libraries with AbS data are few. For this reason, the sequence-specific approach that is based on cDNA libraries from cereal stress resistant genotypes may be improved in different developmental periods to encompass a larger range of tissue types and organs of many species. Functional characterizations of the different AbS treated genes are studied using BlastX [82] by comparing the Swissprot dataset (http://www.uniprot.org/ accessed on 24 April 2021) [83]. Moreover, the EST clustering results provides consensus sequences which are more informative than typical EST data. Drought-responsive genes have a lot of features that include metallothionein-like proteins, late embryonic abundant (LEA) proteins, heat-shock proteins, cytochrome P450 enzymes, catalases, peroxidases, kinases, phosphatases, and TFs (DREB, MYB, MYC, AP2-EREBP, ZF-HD, NAC, WRKY, and bHLH protein) that were abundantly expressed during drought stress [78,84]. Recent research was carried out to identify ESTs in the NaCl-treated cDNA library of *Thellungiella halophila* and also from monocots like barley, wheat, maize, and rice [85]. Furthermore, novel studies are needed to be conducted on all food crops for future food security and production.

## 6. Transcript Signature Usage in Stress Responsive Gene Mining

Several computational approaches are employed to quantify the expression intensities of EST on its programs in cereal crops such as rice [86], barley [87], and maize [88]. The microarray technology is used for transcript expression profiling, Mission Planning and Scheduling System (MPSS), Serial Analysis of Gene Expression (SAGE), and quantitative real-time PCR (qRT-PCR) these are further modern approaches for the quantification under the controlled and stressed tissues of large number of gene expression studies [89]. Microarray-based transcript profiling was carried out in *Arabidopsis* [90] as well as in important cereal species [91,92] have comprehensively analyzed the gene expression signature in response to unique and multiple AbS. An EST-cDNA array technology provides a pivotal tool to compare the relative expression levels between normal and treated plants to unveil the functions of AbS responsive candidates. In addition to that, it is used to identify and understand the genes involved in transcriptional reprogramming and signal transduction pathways.

TFs, are the protein-containing domains that imply the DNA binding and transcript regulatory activities. The TF families were classified into more than 50, based on the presence of highly conserved motifs (Table 2). Computational omics studies were used to identify and understand the molecular cross-talks of plant developmental stages, expression profiling, and physicochemical properties [84,93]. The TFs are mainly involved in diverse stress tolerance and plant growth metabolisms such as cold, salinity, drought, metal, submergence, heat, low/high temperature, light, UV, O_3_, osmotic, oxidative stress, and signaling, tissues development, regulation of plant physiological metabolisms respectively. It is the essential key factor to unravel the candidate gene functions and their dynamism.

Diverse literary information revealed the importance of plant stress mechanisms analyzed by gene expression signature. Two kinds of pathways involved in this type of gene expression studies are (i) desiccation tolerance in an ABA-dependent manner by ABA-responsive element binding factors (ABF), MYC and MYB TFs, and (ii) ABA-independent and associated with drought-responsive element binding factors (DREB) [96]. Recent studies are providing evidence, that other than the ABA-associated pathways, there exists an interlinked relationship between cold and salinity stress signaling pathways.

In cereal crop species, microarray and RNA-Seq based stress-regulated transcripts were used for large spectrum gene expression signature analysis in rice, up-regulation of few candidates that encodes cell division, 40S ribosomal proteins, glycine-rich proteins, elongation factor, and induce the phytohormone regulating genes. The genes which are generally downregulated in the sensitive rice cultivators are glycine-rich proteins, ABA and stress-induced proteins, metallothionein-like proteins, glutathione S-transferase, ascorbate peroxidase, water channel protein isoforms, subtilisin inhibitor, tyrosine inhibitor, and so on [97,98].

During long-term AbS treatments, protease inhibitors, stress proteins, aquaporins, antioxidant components, and some unknown genes were induced and are expected to impart tolerance. A few transcriptional expression signature analyses were conducted in important cereal crops. In *Arabidopsis*, the multiple stress interactions were studied using functional genomics approaches [10,85]. Multiple stress interactions of AbS treatments were investigated to identify the key players having a pivotal role in multiple individual stresses such as drought, cold, flood, salt, UV, and temperature responses [99]. Using 1300 full-length clones, only 44 genes were identified, which are directly induced either by drought or cold stress dynamisms were reported [100]. By using 7000 whole clone inserts, 213 salinity responsible genes, 299 drought responsive genes, 245 ABA-regulating key genes, and 54 cold-regulating genes were identified [101]. Functional omics and bioinformatics tools to identify gene expression patterns related to multiple stress interactions have been considered as a significant aspect of modern plant stress biology research.

To study gene interaction and downstream elements, the analysis and characterization of the transcriptional responses in knock-out mutants or transgenic plants under abiotic stress tolerance are considered a differential method. Three members of the CBF/DREB1 family, CBF1, CBF2, and CBF3 (DREB1b, DREB1c, and DREB1a respectively) quantification of 41 downstream genes as CBF targets were identified by Fowler and Thomashow, [102]. Comparative genome analysis of the AbS responses among diverse tolerant species is extensively considered as an important approach to reconnaissance of evolutionarily conserved and unique stress defense mechanisms [103]. Various promoters of a group of the abiotic stress-responsive genes from different species of maize, rice, and *Arabidopsis* harboring DRE, GCC-box, ABF, and w-box *cis*-elements were reported [104]. Computational omics gene mining and profiling led to the novel way to understand the huge number of genes involved in AbS responses given in Table 3.

## 7. Identification of Genes and Their Agronomic Traits

A case study has been carried out on the evaluation of seven well-known candidate genes for their effects on improving drought resistance of transgenic rice under field conditions [132]. Several AbS affects the growth and yield of cereal crops such as drought, extremely high temperature, low water availability, mineral deficiencies or toxicities, and salinity [133,134]. In recent years, the initiative of developing drought-resistant cereal crops has been well recognized as the most promising and effective strategy for food security against drought and water deficit. In addition, over-expression of certain stress-responsive genes or TFs regulating the multiple stress proteins were shown to confer the increased tolerance to drought as well as in salinity and freezing stresses [117]. Poor management of agricultural water resources, soil degradation, and community pressures are all the prominent stressors that play a pivotal role in the agriculture perspectives across the world. Extensive genetic studies have indicated a huge variation for AbS tolerance but it has failed to attain its maximum goal due to the relatively poor knowledge from the molecular basis for stress-tolerant cereal crop plants. In this case, functional omics and bioinformatics play a crucial role by providing several tools for dissecting AbS responses in cereal crops especially in rice, barley, and wheat through interrogation of genes that may be useful for improving resistance to AbS.

Xiao et al. [132] have selected seven genes (*CBF3*, *SOS2*, *NCED2*, *NPK1*, *LOS5*, *ZAT10*, and *NHX1*) in drought resistance breeding and transformed them into rice cultivar Zhonghua 11 (*O. sativa* L. ssp. *Japonica*) under the control of a constitutive promoter (from rice *Actin1* gene) and an inducible promoter (from a rice homologous gene of *HVA22*), respectively. The developed transgenic rice was responsible for drought resistance under natural field conditions. These traits (Table 4) are an example that can be a useful reference for drought resistance engineering in cereal crops and also pave the way to address the other stress-related issues.

## 8. Genome-Wide Association Studies (GWAS)

By the use of an ultra- high throughput genotyping technology, GWAS became available as a powerful alternative for dissecting quantitative traits in crop plants [149]. GWAS provides an understanding of transcriptional regulation, metabolic response of rice and other cereal crops to diverse environmental conditions and also in significant relation to AbS [150]. Genome-wide association mapping identifies major QTL regions without the need of constructing a mapping population.

Currently, GWAS was categorized into two types, population-based association studies and a family-based approach [151]. In population-based association studies, it was revealed that unrelated individuals are used to examine genome-wide associations between single nucleotide polymorphisms (SNPs) and their associated phenotypic traits. Family-based mapping studies can be applied to complex pedigrees derived from the crosses among different genotypes. Both approaches have complementary pros and cons. Population-based GWAS takes advantage of more recombination events that have accumulated over time of generations in historical populations, with the disadvantage of finding false positives or false negatives results applied in *Arabidopsis* [152]. Family-based GWAS can eliminate the effects of population structure and therefore escape the false- positives and false-negatives, but recombination accumulated over a few generations during pedigree development used in *Z. mays* NAM (nested association mapping) population was developed to characterize flowering regulation [151]. The maize NAM population of 200 recombinant inbred lines from 25 parents were crossed to the fully sequenced genotype (B73). Therefore, GWAS is an alternative and complementary approach for understanding the trait and molecular level mechanisms in plants. This approach paves the un-paralleled way for the history of plant stress and molecular biology.

## 9. Conclusions and Future Perspectives

Ever-increasing advances in multiple analytical stages are paving the way to address the diverse omics scale outcomes, fortifying the researcher’s knowledge and long-standing questions in plant biology. Plants are acclimatizing to the environment by altering their genome, metabolome, transcriptome, lipidome, proteome, secretome and miRNAome. Even in this post-genome era, the complete understanding of plant molecular responses to the stress tolerance mechanism is not yet achieved, as the list of genes involved in stress response is increasing rapidly. The identity of these proteins and their functions are close to being determined. Post-transcriptional studies including splicing and RNA silencing and posttranslational mechanisms such as SUMOylation, phosphorylation, and ubiquitination lead to a prompt response in plants against stress. Thus, employing the developing omics approaches and GWAS will contribute to better understanding the AbS response.

At the same time, we are majorly facing multiple global issues such as climate changes, global warming, water, food, and energy security. Further, voluminous research is highly needed to unravel the specific molecular function of the plants. AbS tolerance cross-talks that are speculating in particular plant species, significantly in C_3_ and C_4_ grass species, are also essential in identifying the candidates to improve the diverse molecular and biochemical functions. In addition, these molecular studies are also subjected and compared to advanced omics datasets from C_3_ and C_4_ genome species, which are helping to improve the applied or translational research, to unveil the plant molecular systems in response to stress biologist, molecular biologist, and plant physiologist, and also the ever-increasing command of mankind. This eagle’s eye review can open the penstocks to budding scientists.

## Figures and Tables

**Table 1:** Comparative nutritive value of cereal grains.

Factor	Wheat	Maize	Rice	Barley	Sorghum	Oat	Millet	Rye
Available CHO (%)	69.7	63.6	64.3	55.8	62.9	62.9	63.4	71.8
Energy (kJ/100 g)	1570	1660	1610	1630	1610	1640	1650	1570
Digestible energy (%)	86.4	87.2	96.3	81.0	79.9	70.6	87.2	85.0
**Vitamins (mg/100 g)**								
Thiamin	0.45	0.32	0.29	0.10	0.33	0.60	0.63	0.66
Riboflavin	0.10	0.10	0.04	0.04	0.13	0.14	0.33	0.25
Niacin	3.7	1.9	4.0	2.7	3.4	1.3	2.0	1.3
**Amino acids (g/16 g N)**								
Lysine	2.3	2.5	3.8	3.2	2.7	4.0	2.7	3.7
Threonine	2.8	3.2	3.6	2.9	3.3	3.6	3.2	3.3
Met. & Cys.	3.6	3.9	3.9	3.9	2.8	4.8	3.6	3.7
Tryptophan	1.0	0.6	1.1	1.7	1.0	0.9	1.3	1.0
**Protein quality (%)**								
True digestibility	96.0	95.0	99.7	88.0	84.8	84.1	93.0	77.0
Biological value	55.0	61.0	74.0	70.0	59.2	70.4	60.0	77.7
Net protein utilization	53.0	58.0	73.8	62.0	50.0	59.1	56.0	59.0
Utilization protein	5.6	5.7	5.4	6.8	4.2	5.5	6.4	5.1

**Table 2:** C3 and C4 crop species transcription factor families and their number of family members. (Source: https://grassius.org/grasstfdb.php/ accessed on 19 May 2021) [94] (http://planttfdb.cbi.pku.edu.cn/ accessed on 19 May 2021) [95]).

S. No	Transcription Factors (TFs)	Rice	Sorghum	Maize	Wheat
1	ABI3-VP1	55	60	51	-
2	BBR/BPC	04	05	04	05
3	C2C2-GATA	23	27	36	-
4	CCAAT-HAP2	11	09	16	-
5	G2-like	44	41	59	100
6	HSF	25	24	29	53
7	Orphans	185	177	339	-
8	WHIRLY	02	03	02	07
9	Alfin-like	09	13	19	-
10	bHLH	135	143	175	-
11	C2C2-YABBY	08	08	13	-
12	CCAAT-HAP3	12	-	04	-
13	GeBP	13	15	21	12
14	MADS	70	76	77	-
15	SBP	19	18	32	37
16	WRKY	103	94	125	171
17	AP2-EREBP	164	156	212	-
18	bZIP	92	91	128	186
19	C2H2	09	07	10	224
20	CCAAT-HAP5	22	-	18	-
21	GRAS	60	76	86	121
22	MYB	114	113	167	263
23	TCP	22	28	44	28
24	ZF-HD	15	15	21	20
25	ARF	27	27	38	45
26	BZR	06	08	10	-
27	C3H	46	44	54	100
28	CPP	11	08	13	24
29	GRF	12	10	15	16
30	MYB-related	71	80	116	227
31	Trihelix	17	19	43	46
32	ZIM	21	19	36	-
33	ARID	06	07	10	-
34	C2C2-CO-like	08	09	14	-
35	CAMTA	06	06	05	20
36	E2F-DP	09	10	19	24
37	Homeobox	95	83	133	-
38	NAC	144	123	134	263
39	TUB	15	13	15	-
40	ARR-B	06	10	08	22
41	C2C2-Dof	30	29	46	-
42	CCAAT-DR1	01	-	17	-
43	EIL	09	07	09	16
44	HRT	01	01	-	-
45	NLP	13	13	17	-
46	VOZ	02	02	05	06
47	CCAAT	-	32	-	-
48	mTERF	-	-	30	-
49	OVATE	-	-	43	-
50	Sigma70-like	-	-	09	-
51	PLATZ	-	-	15	-
52	FAR1-like	-	-	15	-
53	Rcd1-like	-	-	10	-
54	FLO/ LFY	-	-	02	-
55	S1Fa-like	-	-	02	03
56	CSD	-	-	04	-
57	LBD	-	-	44	61
58	DBP	-	-	04	-
59	SHI/STY (SRS)	-	-	09	-
60	AP2	-	-	-	43
61	BES1	-	-	-	10
62	ERF	-	-	-	181
63	HRT-like	-	-	-	03
64	M-type-MADS	-	-	-	77
65	NF-X1	-	-	-	02
66	RAV	-	-	-	08
67	TALE	-	-	-	52
68	DBB	-	-	-	17
69	FAR1	-	-	-	201
70	MIKC_MADS	-	-	-	51
71	NF-YA	-	-	-	22
72	Dof	-	-	-	52
73	HB-PHD	-	-	-	06
74	NF-YB	-	-	-	34
75	YABBY	-	-	-	25
76	B3	-	-	-	140
77	GATA	-	-	-	48
78	HB-other	-	-	-	44
79	LFY	-	-	-	02
80	NF-YC	-	-	-	20
81	SRS	-	-	-	05
82	CO-like	-	-	-	07
83	HD-ZIP	-	-	-	62
84	LSD	-	-	-	13
85	Nin-like	-	-	-	29
86	STAT	-	-	-	02
87	WOX	-	-	-	26

‘-’ indicates that TF family was absent in particular crop.

**Table 3:** Genes encoding enzymes/proteins associated with abiotic stress response in cereals.

Gene Category	Gene	Cellular Response	Species	Reference
**Osmolyte compounds**				
Glycine betaine	*BADH*	Heave metal stress	Rice	[105]
	*CodA*	Salt, Cold and drought stress	Rice	[106]
	*bet A*	Cold, Drought stress	Maize	[107]
Proline	*P5CS*	Drought	Wheat	[108]
**Regulatory genes**				
bZIP	*bZIP4*	Salinity stress	Maize	[109]
	*HBP1b*	Drought, Salt, cold	Rice	[110]
	*bZIP16*	Dehydration, salt and ABA	Rice	[111]
**Transporters**				
Na+-H+-dependent K+ transporter	*ZmHKT1*	Salt stress	Maize	[112]
Na+-K+-symporter	*HKT1*	Salt stress	Wheat	[113]
	*HKT1*	Salt stress	Rice	[114]
**Stress-responsive genes**				
Transcription factors	*SAP7*	Abiotic stress	Rice	[115]
	*DREB*	Abiotic stress	Maize	[116]
	*MYB6*	Drought and Salt	Rice	[117]
WCS genes	*WCS19*	Cold stress	Wheat	[118]
	*WCS120*	Cold stress	Wheat	[118]
Thaumatin-like protein	*TLP14*	Cold stress	Barley	[119]
Heat shock protein	*HSFA7*	Salt and Droght	Rice	[120]
	*HSP20*	Heat stress	Wheat	[121]
RAB genes	*RAB7*	Drought and Heat stress	Rice	[122]
	*RAB11*	Salt stress	Rice	[123]
LEA proteins	*HVA1*	Drought stress	Barley	[124]
	*HVA1*	Salt, Cold and dehydration stress	Rice	[125]
	*HVA1*	Salt and Drought tolerance	Barley	[126]
**Antioxidants**				
Ascorbate peroxidase	*APX*	Drought, Salt and Cold	Rice	[127]
Catalase	*CAT*	Drought stress	Wheat	[128]
Superoxide dismutase	*MnSOD*	Abiotic stress	Rice	[129]
	*ZnSOD*	Salt stress	Rice	[130]
	*FeSOD*	Drought stress	Rice	[131]

**Table 4:** Transgenic crop plants developed for AbS tolerance details.

Gene	Cellular Response	Species	Reference
**Osmolyte compounds**			
**Pyrroline carboxylate synthase (p5cs)**	Drought tolerance	Wheat	[108]
**Choline dehydrogenase**	Drought, Salt tolerance	Rice	[135]
**Arginine decarboxylase**	Drought, Heat, Freezing, Salinity tolerance	Rice	[136]
**Glutamine synthetase**	Oxidative stress tolerance	Rice	[137]
**Trehalose-6-P-synthase**	Salt, Drought, Cold tolerance	Rice	[138]
**Mannitol dehydrogenase**	Drought, Salt tolerance	Wheat	[139]
**Regulatory genes**			
**Calcium dependent protein kinase**	Drought tolerance	Rice	[140]
**DREB1A**	Drought tolerance	Rice	[141]
**Transporters/symporter**			
**Na+/H+ antiporter**	Salt tolerance	Rice	[142]
**Potassium transporter (HKT1)**	Salt tolerance	Rice	[143]
**Stress-responsive genes**			
**HVA1**	Drought, Salt tolerance	Rice	[125]
**Alcohol dehydrogenase**	Submergence tolerance	Rice	[144]
**Ferritin**	Heat tolerance	Wheat	[145]
**HVA1**	Salt and Drought tolerance	Barley	[126]
**Pyruvate decarboxylase1**	Submergence tolerance	Rice	[146]
**Antioxidants**			
**Fe-superoxide dismutase**	Drought tolerance	Rice	[147]
**Mn-superoxide dismutase**	Salt tolerance	Rice	[148]

## Data Availability

Not applicable.
